# Regulation of epithelial-mesenchymal transition by protein lysine acetylation

**DOI:** 10.1186/s12964-022-00870-y

**Published:** 2022-04-28

**Authors:** Fanyun Kong, Lihong Ma, Xing Wang, Hongjuan You, Kuiyang Zheng, Renxian Tang

**Affiliations:** 1 Jiangsu Key Laboratory of Immunity and Metabolism, Department of Pathogenic Biology and Immunology, Xuzhou Medical University, Xuzhou, Jiangsu China; 2 National Demonstration Center for Experimental Basic Medical Sciences Education, Xuzhou Medical University, Xuzhou, Jiangsu China

**Keywords:** Epithelial-mesenchymal transition, Lysine acetylation, Histone, Non-histone protein, Therapy

## Abstract

The epithelial-mesenchymal transition (EMT) is a vital driver of tumor progression. It is a well-known and complex trans-differentiation process in which epithelial cells undergo morphogenetic changes with loss of apical-basal polarity, but acquire spindle-shaped mesenchymal phenotypes. Lysine acetylation is a type of protein modification that favors reversibly altering the structure and function of target molecules via the modulation of lysine acetyltransferases (KATs), as well as lysine deacetylases (KDACs). To date, research has found that histones and non-histone proteins can be acetylated to facilitate EMT. Interestingly, histone acetylation is a type of epigenetic regulation that is capable of modulating the acetylation levels of distinct histones at the promoters of EMT-related markers, EMT-inducing transcription factors (EMT-TFs), and EMT-related long non-coding RNAs to control EMT. However, non-histone acetylation is a post-translational modification, and its effect on EMT mainly relies on modulating the acetylation of EMT marker proteins, EMT-TFs, and EMT-related signal transduction molecules. In addition, several inhibitors against KATs and KDACs have been developed, some of which can suppress the development of different cancers by targeting EMT. In this review, we discuss the complex biological roles and molecular mechanisms underlying histone acetylation and non-histone protein acetylation in the control of EMT, highlighting lysine acetylation as potential strategy for the treatment of cancer through the regulation of EMT.

**Video Abstract**

**Supplementary Information:**

The online version contains supplementary material available at 10.1186/s12964-022-00870-y.

## Background

The epithelial-mesenchymal transition (EMT) is a well-known, complex, reversible cellular trans-differentiation process. During EMT, epithelial cells are not only capable of reorganizing the cytoskeleton and losing cell–cell contacts, but also gain a mesenchymal phenotype to facilitate cell motility, resistance to apoptosis, and ECM production [1, 2]. A strong association between EMT and embryonic organ development, tissue fibrosis, wound healing, and the progression of various cancers has been reported [3, 4]. EMT can regulate tumor migration, chemotherapeutic resistance, metastasis, and the acquisition of stem cell-like characteristics [1, 4]. Moreover, when epithelial cells undergo EMT, the expression of epithelioid markers, including E-cadherin, claudin-1, and EpCAM, is repressed. In contrast, mesenchymal markers, such as N-cadherin, αSMA, and vimentin, are upregulated during this transition [4–7]. In addition, current evidence shows that EMT is a stepwise complex process with distinct intermediate states rather than functioning in a binary manner [8, 9]. In particular, preclinical and clinical observations have demonstrated that some cancer cells undergoing EMT only exhibit partial EMT or a hybrid epithelial/mesenchymal (E/M) state combining both epithelial and mesenchymal phenotype [10–12]. Therefore, further clarification of the hybrid E/M status of different tumors with EMT and the associated molecular mechanisms is needed to provide promising biomarkers for the diagnosis, prognosis, and treatment of various cancers [10].

Similar to many other biological programs, EMT is controlled by a series of EMT-inducing transcription factors (EMT-TFs), in particular by three main families of EMT-TFs, namely Snail (Snail1/Snail and Sanil2/Slug), Twist (Twist1 and Twist2), and ZEB (ZEB1 and ZEB2), to orchestrate gene expression, including the restriction of epithelial genes and sensitization of mesenchymal genes [13]. In addition, many signaling pathways, including JAK/STAT, NF-κB, TGF-β/Smad, and PI3-K have been demonstrated to be essential for the induction of EMT [4, 14]. More importantly, computational and experimental evidence shows that different types of epigenetic modulation, depending on chromatin alterations [15, 16], DNA methylation, histone modifications [17], and non-coding RNA [18–20], play a vital role in modulating the expression levels of EMT-associated genes by regulating chromatin accessibility, transcription factor activity, and the promoter status of target genes. In addition, post-translational modifications (PTMs), including ubiquitylation, phosphorylation, and sumoylation, can also control EMT by adjusting protein stability, intracellular localization, protein structure, and the function of EMT-related molecules [21].

Lysine acetylation, a conserved protein modification, can transfer an acetyl group from acetyl-coenzyme A to lysine residues on target molecules to change their structures and biological functions [22]. Although acetylation was first discovered in histones 50 years ago [23], advances in proteomics in the last decade have indicated that non-histone proteins can also be acetylated [24]. Importantly, the acetylation or deacetylation levels in most of the identified histone and non-histone proteins result from the balance of opposing enzymatic activities between lysine acetyltransferases (KATs, also called histone acetyltransferases, HATs) and lysine deacetylases (KDACs, also called histone deacetylases, HDACs). Mammalian KATs are divided into nuclear and cytoplasmic KATs, according to their cellular localization. Nuclear KATs are classified into five families based on sequence similarities, including basal transcription factors, GCN5/PCAF, MYST, CBP/p300, and the nuclear receptor coactivator family. However, only a few cytoplasmic KATs have been identified, including KAT1 and TAT1. In addition, KDACs are divided into four classes: class I (HDAC1-3, 8) [22], class II (HDAC4-6, HDAC9-10) [23], class III sirtuins (SIRT1-7) [24], and class IV (HDAC11) [25, 26]. The effects of acetylation on histones and non-histone proteins vary in various biological processes. In particular, histone acetylation is linked to the control of target gene transcription [22], while non-histone protein acetylation contributes to the modulation of enzymatic activity, protein stability, and subcellular localization [24]. Moreover, accumulating evidence has demonstrated that the acetylation of histones and non-histone proteins is essential for EMT.

In this review, we discuss evidence on the biological role and molecular mechanisms associated with both histone and non-histone protein acetylation to control EMT and the potential of targeting protein acetylation to inhibit EMT, which is beneficial for cancer treatment.

### Contribution of histone acetylation to EMT regulation

Epigenetics mostly regulates all biochemical processes by alternating the genome without changing the nucleotide sequence [18]. To date, different epigenetic changes mediated by non-coding RNAs, DNA methylation, and histone modifications have been identified in the process of EMT [18, 27]. Although the current evidence shows that DNA methylation and histone modification can influence the transcription of target genes, the non-coding RNAs have the capability of regulating the expression of target genes at the post-transcriptional level [15–20], our information regarding epigenetic gene regulation still remains limited. Recent studies have shown that epigenetic molecules can be used for the diagnosis, prediction of prognosis, and therapy response in cancer patients [18]. In addition, understanding the mechanisms responsible for epigenetics-mediated EMT is vital for the discovery of novel strategies to prevent EMT-related cancer progression. Histone acetylation, as a type of epigenetic modification, has a prominent effect on the opening of chromatin assembly and activation of target gene transcription. However, histone deacetylation plays the opposite role [22, 23]. In particular, many studies have revealed that acetylation of distinct histones, including histone 2 (H2) [28, 29], histone 3 (H3) [30, 31], and histone 4 (H4) [32], at the promoter regions of certain genes, is crucial for the regulation of EMT. EMT marker genes, EMT-TFs, and EMT-related long non-coding RNAs (lncRNAs) mediated by histone acetylation have been reported to benefit EMT (Table 1).

**Table 1:** The information on histone acetylation to regulate EMT-related factors

Target molecule	Molecular type	The expression levels of target molecules during EMT	The regulator of target molecule	Histone modifier	Acetylation sites	Target cells	References
E-cadherin	EMT-related epithelioid marker	Down	miR-N5	CBP	H3K56	Prostate cancer cells	[33]
E-cadherin	EMT-related epithelioid marker	Down	SIRT1	SIRT1	H4K16	Prostate cancer cells	[34]
E-cadherin	EMT-related epithelioid marker	Down	Snail1	HDAC1, HDAC2	H3, H4	Kidney cells	[35]
E-cadherin	EMT-related epithelioid marker	Down	Snail2	HADC1, HADC2, HADC3	H3K4, H3K56	Lung carcinoma cells	[36]
E-cadherin	EMT-related epithelioid marker	Down	ZEB1	HDAC1, HDAC2	H3, H4	Pancreatic cancer cells	[37]
EpCAM	EMT-related epithelioid marker	Down	ZEB1	unknown	H3K9, H3K27, H4	Lung cancer cells	[38]
E-cadherin	EMT-related epithelioid marker	Down	CPEΔN	HDAC1, HDAC3	H3K9	Lung cancer cells	[39]
E-cadherin	EMT-related epithelioid marker	Down	TRIM28	unknown	H3K9	Lung cancer cells	[40]
E-cadherin	EMT-related epithelioid marker	Down	HOTAIR	CBP	H3K27	Gastric cancer cells	[41]
αSMA	EMT-related mesenchymal marker	Up	TGF-β1	unknown	H4	Lens epithelial cells	[42]
αSMA	EMT-related mesenchymal marker	Up	TGF-β2	unknown	H3K27	Kidney cells	[43]
N-cadherin	EMT-related mesenchymal marker	Up	Ajuba, Twist1	CBP	H3	Colorectal cancer cells	[44]
vimentin	EMT-related mesenchymal marker	Up	unknown	unknown	H3	Prostate cancer cells	[45]
Snail1	EMT-TF	Up	DOT1L	P300	H3	Breast cancer cells	[46]
Snail2	EMT-TF	Up	SND1	GCN5, P300,	H3K9, H3K14, H3K18	Ovarian cancer cells	[48]
Snail2	EMT-TF	Up	KLF10	HDAC1	H3K9, H3K27	Lung adenocarcinoma cells	[49]
Snail2	EMT-TF	Up	TGF-β	HDAC1, HDAC3	H3K56, H3K4	HCC cells	[50]
Snail2	EMT-TF	Up	LncRNA RP11-367G18.1	unknown	H4K16	In head and neck cancer cells	[51]
Twist 1	EMT-TF	Up	HAUSP, HIF-1a	unknown	H3K56 a	Multiple cancer cells	[52]
Twist 1	EMT-TF	Up	Wnt/β-catenin, PI3-K signalings	unknown	H3K27	Gastric cancer cells	[53]
Twist 1	EMT-TF	Up	LncRNA RP11-367G18.1	unknown	H4K16	In head and neck cancer cells	[51]
Twist2	EMT-TF	Up	ACOT12	GCN5	H3	HCC cells	[54]
ZEB1	EMT-TF	Up	MEF2D	P300	H3, H4	Colorectal cancer cells	[55]
ZEB2	EMT-TF	Up	unknown	unknown	H3	Prostate cancer cells	[45]
ZEB1	EMT-TF	Up	DOT1L	P300	H3	Breast cancer cells	[46]
ZEB2	EMT-TF	Up	DOT1L	P300	H3	Breast cancer cells	[46]
GHET1	EMT-related LncRNA	Up	unknown	unknown	H3K27	HCC cells	[56]
ROR	EMT-related LncRNA	Up	CBP	CBP	H3K27	Retinoblastoma cells	[57]
TINCR	EMT-related LncRNA	Up	CBP	CBP	H3K27	Breast cancer cells	[58]
PLAC2	EMT-related LncRNA	Up	CBP	CBP	H3K27	Oral squamous cell carcinoma cells	[59]
ANCR	EMT-related LncRNA	Up	HDAC3	HDAC3	H3, H4	HCC cells	[32]

### Histone acetylation in modulating the promoters of EMT marker genes

During EMT, different cancer cells undergo complicated morphogenetic changes with a decrease in epithelial markers, including E-cadherin and EpCAM [3, 7]. In particular, evidence shows that the modulation of histone acetylation at the promoters of E-cadherin and EpCAM stimulated by multiple cellular factors participates in the control of the expression of these two molecules to facilitate EMT. According to the results of chromatin immunoprecipitation (ChIP) assays, miR-N5 decreases CBP, a component of KATs, and mediates H3K56 deacetylation at the promoter of E-cadherin to suppress its expression in prostate cancer [33]. Based on ChIP-coupled quantitative polymerase chain reaction (ChIP-qPCR) experiments, SIRT1, a component of KDACs, was found to induce the deacetylation of H4K16, which is essential for E-cadherin silencing to facilitate EMT in prostate cancer cells [34].

As two core EMT-TFs, Snail1 and Snail2 are often upregulated and have the capability of inhibiting the expression of E-cadherin through a variety of molecular mechanisms, including histone acetylation, to facilitate cancer progression. It is worth noting that Snail1 can bind to the promoter of E‑cadherin to trigger chromatin changes with a loss of H3/H4 acetylation at its promoter and downregulate the expression of the E-cadherin gene. Mechanistically, based on ChIP and co-immunoprecipitation (Co-IP) assays, Snail1 has been shown to interact with and recruit two members of HADCs, HDAC1 and HDAC2, to the E-cadherin promoter to facilitate H3 and H4 deacetylation in kidney cells [35]. In addition to Snail1, the inhibition of E-cadherin mediated by Snail2 has been observed to be related to the deacetylation of both H3K4 and H3K56 at the promoter of E-cadherin in lung carcinoma cells [36]. HADC1, HADC2, and HADC3 was found to interact with Snail2 and participate in the regulation of histone acetylation at the E-cadherin promoter. ZEB1, another well-known EMT-TF, recruits HDAC1 and HDAC2, as determined by ChIP and Co-IP assays, to the promoter of E-cadherin to inhibit H3 and H4 acetylation and then downregulate its expression in pancreatic cancer cells [37]. ZEB1 also induces downregulation of EpCAM. In particular, following ZEB1 regulation, the acetylation levels of H3K9, H3K27, and H4 are decreased at the EpCAM promoter, as detected by ChIP experiments, and these reduced histone acetylation have a predominant role in EpCAM inhibition in lung cancer cells [38]. However, the KATs responsible for histone acetylation regulated by ZEB1 to control EpCAM have yet to be fully elucidated.

In addition, the N-terminal-truncated carboxypeptidase E (CPEΔN) protein is a crucial regulator of lung cancer metastasis. Sun et al. showed that CPEΔN accelerated EMT in lung cancer cells. Mechanistically, the CPEΔN protein can cause a reduction in histone H3K9 acetylation at the E-cadherin promoter, which was examined by ChIP assay to block its transcription [39]. HDAC1 and HDAC3 maybe participate in the regulation of histone H3K9 acetylation mediated by CPEΔN. Chen et al. showed that TRIM28 is implicated in TGF-β-stimulated EMT. E-cadherin was upregulated, while Snail1, Snail2, and Twist1 were decreased in TRIM28 knockdown cells. Moreover, elevated deacetylation of H3K9 mediated by TRIM28 is related to the decline of E-cadherin in lung cancer cells [40]. Until now, the molecular mechanisms that contribute to the modulation of histone acetylation at the E-cadherin promoter mediated by TRIM28 have not been fully elucidated, and are worth exploring in the future.

LncRNAs are non-coding RNAs of > 200 bp. Evidence shows that lncRNAs play crucial regulatory roles in EMT in various human cancers. As an important lncRNA, HOX transcript antisense intergenic RNA (HOTAIR) contributes to the development of multiple cancers. In particular, Song et al. showed that, based on the UCSC Genome Browser and ChIP assay, HOTAIR is found to facilitate the decrease in histone H3K27 acetylation at the E‑cadherin promoter to inhibit its expression and promote EMT in gastric cancer cells. In addition, decreased histone H3K27, mediated by HOTAIR, has been closely correlated with CBP inhibition (Table 1) [41]. In addition to HOTAIR, whether other lncRNAs participate in the modulation of histone acetylation to regulate E-cadherin expression is unknown. Furthermore, although factors, such as EMT-TFs, CPEΔN, and lncRNA, as mentioned above, facilitate the inhibition of E-cadherin based on the modulation of histone acetylation, whether these factors affect histone acetylation at the promoters of other EMT epithelioid markers is not elaborated and requires further investigation.

Moreover, accumulating evidence has demonstrated that EMT is also related to elevated histone acetylation at the promoters of EMT-related mesenchymal markers, including αSMA, N-cadherin, and vimentin, to facilitate their upregulation in cancers. A recent study showed that the increased acetylation of histone H4 at the αSMA promoter is associated with EMT stimulated by TGF-β2, as detected by the ChIP assay in lens epithelial cells [42]. In addition, TGF-β1 stimulation also increases the recruitment of acetylated H3K27 to the promoters of α-SMA in kidney cells [43]. Twist1 is a core EMT transcription factor that contributes to the transcription of N-cadherin, an EMT-related mesenchymal marker. Relying on Co-IP and ChIP experiments, Wu et al. showed that Ajuba, a multiple LIM domain-containing protein, can recruit CBP as well as Twist1 to form a protein complex at the Twist1-binding region and enhance the acetylation of histone H3 at the N-cadherin promoter in colorectal cancer cells [44]. In addition, increased acetylation of histone H3 in the vimentin promoter, as measured by the ChIP assay, is related to the elevation of the gene in prostate cancer cells [45]. However, the mechanisms related to the increase in histone acetylation at the promoters of these mesenchymal markers remain unknown.

### Histone acetylation in modulating the promoters of EMT-TFs

The EMT-TFs Snail, Twist, and ZEB, which are increased and activated early in the process of EMT, have been found to play central roles in the development of different cancers [3]. Importantly, recent studies have shown that the elevation of EMT-TFs mediated by histone acetylation with stimulation by different cellular factors, has beneficial effects on EMT. The roles of histone acetylation and the associated molecular mechanisms of EMT regulation by EMT TFs are described below.

### Snail1 and Snail2

As two core EMT-TFs, Snail1 and Snail2 are increased and activated early and sufficiently to initiate EMT [13, 27]. The histone H3 acetylation of the Snail1 promoter is associated with the regulation of Snail1 expression. Moreover, based on ChIP-qPCR analysis, the recruitment of P300 mediated by DOT1L was found to be related to acetylation of H3 at its promoter in breast cancer cells [46]. Notably, histone acetylation at the promoters of Snail2 plays an essential role in its expression to accelerate EMT. For instance, increased histone H3 acetylation is related to elevated Snail2 expression in breast cancer cells [47]. Among the KATs, both GCN5 and P300 have been shown to contribute to histone H3K9, H3K14, and H3K18 acetylation at the Snail2 promoter, as examined by ChIP analysis. However, loss-of-function of SND1 results in a reduced recruitment of GCN5 and P300 to the Snail2 promoter, leading to a reduction in H3K9, H3K14, and H3K18 acetylation at its promoter to inhibit EMT in ovarian cancer cells [48]. Among KDACs, HDAC1 and HDAC3 suppress Snail2 expression by inhibiting histone acetylation at its promoter. For example, based on ChIP-seq and ChIP analyses, Mishra et al. found that the transcription factor KLF10 occupying GC-rich sequences at the promoter of Snail2 could repress Snail2 transcription by recruiting HDAC1, which blocked the acetylation of H3K9 and H3K27 at the promoter of Snail2 in lung adenocarcinoma cells [49]. In addition, during TGF-β-initiated EMT, the effect of TGF-β on Snail2 expression is related to the inhibition of HDAC1 and HDAC3, which can suppress Snail2 transcription by downregulating the acetylation levels of H3K56 and H3K4 in hepatocellular carcinoma (HCC) cells [50]. Hypoxia is a major environmental factor that induces gene reprogramming and initiates EMT. Under hypoxia, lncRNA RP11-367G18.1 plays an essential role in the elevation of H4K16 acetylation at the Snail2 promoter to enhance its expression in head and neck cancer cells (Table 1) [51].

### Twist1 and Twist2

Up to now, Twist1 also has been reported to be regulated by histone acetylation. In particular, based on the ChIP assay, hypoxia was found to induce the polyubiquitination of HAUSP, which stabilizes HIF-1α and then causes H3K56 acetylation at the promoter of Twist1 to accelerate EMT, while H3K56 acetylation mediated by CBP contributes to the elevation of Twist1 transcription in multiple cancer cells [52]. In addition, EMT induced by Wnt/β-catenin and PI3-K signaling is correlated with the acetylation of H3K27 at the Twist1 promoter in gastric cancer cells [53]. LncRNA RP11-367G18.1, induced by hypoxia, also increases the acetylation of H4K16 located on the promoter of Twist1 to facilitate its expression and activation [51]. In addition to Twist1, elevated Twist2 expression was also found to be associated with an increased acetylation of H3 at the Twist2 promoter in HCC cells. Furthermore, the H3 acetylation of its promoter can be inhibited by ACOT12 [54]. Interestingly, the role of ACOT12 in the decrease of H3 acetylation at the Twist2 promoter, detected by the ChIP assay, can be abolished by GCN5 (Table 1).

### ZEB1 and ZEB2

ZEB1 has also been reported to be modulated by histone acetylation, contributing to EMT activation. Su et al. showed that MEF2D directly regulates ZEB1 transcription by promoting histone H3 and H4 acetylation at its promoter to facilitate EMT. With respect to molecular mechanisms, MEF2D significantly increases P300-binding to the promoter of ZEB1, and P300 may account for the upregulation of histone acetylation at the ZEB1promoter in colorectal cancer cells [55]. In addition to ZEB1, increased H3 acetylation is related to increase ZEB2 gene expression in prostate cancer [45]. Moreover, Cho et al. showed that, depending on Co-IP and ChIP analysis, DOT1L cooperates with the c-Myc-P300 complex and initiates histone H3 acetylation at promoters of ZEB1 and ZEB2 (Table 1). Meanwhile, the protein complex results in the dissociation of HDAC1 from the promoters of these genes in breast cancer cells [46].

### Histone acetylation in modulating the promoters of EMT-related LncRNA

As mentioned above, lncRNAs play vital roles in EMT activation. Among the molecular mechanisms associated with the regulation of lncRNA, the increased acetylation of histones at the lncRNA promoters facilitates the upregulation of lncRNA to trigger EMT. For example, elevated H3K27 acetylation at the promoter of lncRNA GHET1 (gene gastric carcinoma highly expressed transcript 1) activates its expression to enhance EMT in HCC cells [56]. Using quantitative reverse transcription PCR (RT-qPCR), the UCSC Genome Browser, and ChIP assays, the expression of lncRNA-ROR was found to be upregulated by the acetylation of histone H3K27 to induce EMT in retinoblastoma cells, and elevated H3K27 acetylation at the lncRNA-ROR promoter was associated with CBP [57]. The expression levels of lncRNA terminal differentiation-induced non-coding RNA (TINCR) significantly increased in trastuzumab-resistant breast cancer cells. The activation of TINCR by H3K27 acetylation positively modulated EMT in breast cancer cells. In addition, H3K27 acetylation in the promoter region of TINCR mediated by CBP has been found to be related to the upregulation of TINCR [58]. Chen et al. showed that LncRNA PLAC2 (placenta-specific protein 2) was upregulated in both oral squamous cell carcinoma cell lines and primary tissue samples. Enriched H3K27 acetylation at the PLAC2 promoter facilitates EMT in oral squamous cell carcinoma. Furthermore, based on ChIP experiments, CBP was shown to increase the acetylation levels of H3K27 at the PLAC2 promoter, thereby upregulating PLAC2 [59]. The lncRNA ANCR has also been found to play vital roles in EMT regulation and tumor metastasis in many tumors. Wen et al. reported that elevated H3/H4 histone acetylation at the ANCR promoter is related to an increase of the lncRNA. Moreover, blocking HDAC3 can increase ANCR expression in HCC cells [32]. Current evidence shows that many lncRNAs participate in the regulation of EMT in different cancers [60–62]. However, the effect of histone acetylation on the regulation of these lncRNAs remains largely unknown.

### Contribution of non-histone acetylation to EMT regulation

**Fig. 1:**
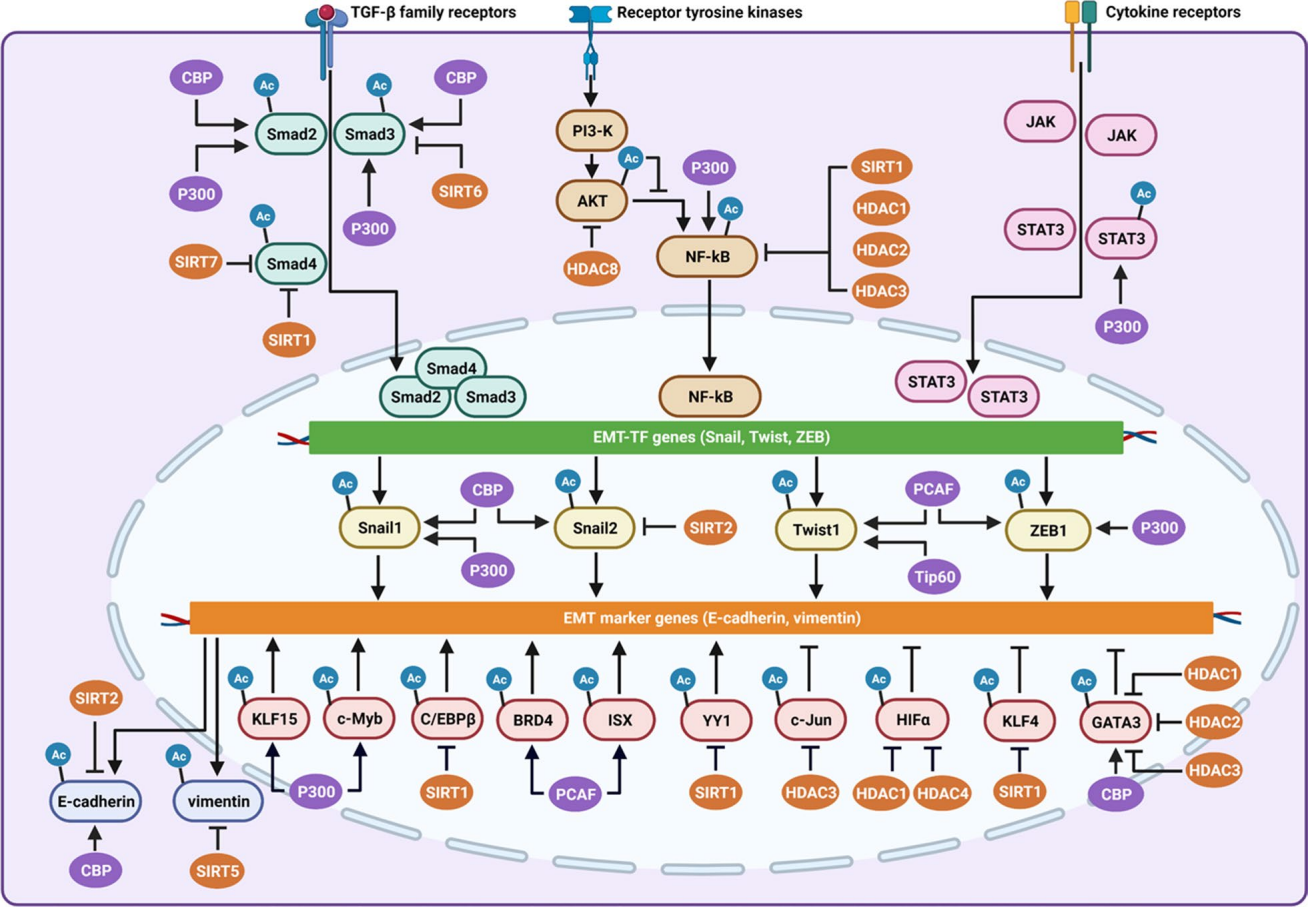
The acetylation of EMT-related cellular factors and their associated KATs and KADCs to initiate EMT. The acetylation of EMT-related signal transduction molecules in TGF-β, PI3-K, and JAK/STAT pathways, to enhance the expression of EMT-TF gene expression, and then acetylation of EMT-TFs, including Snail1, Snail2, Twist1, ZEB1, and other transcription factors, including KLF15, YY1, and c-Jun, to regulate EMT markers expression. Additionally, the modulation of the acetylation of E-cadherin and vimentin can also regulate EMT. The KATs and KADCs, which have identified to contribute to the regulation of the acetylation of EMT-related cellular factors also were added in the Figure. EMT-TFs, EMT-inducing transcription factors, Ac: acetylation

Although histones have been extensively reported to regulate gene transcription to control EMT, recent findings indicate that non-histone proteins could also be frequently acetylated [22, 24]. Moreover, acetylation can affect the subcellular localization, stability, and enzymatic activity of non-histone proteins [24]. To date, the regulation of the acetylation of EMT marker proteins, EMT-TFs, and signal transduction molecules in multiple signaling pathways related to EMT has been investigated (Fig. 1).

### Non-histone acetylation in modulating EMT marker proteins

Among EMT-related epithelioid markers, E-cadherin is mainly located on the cell surface and is downregulated during EMT. To date, the nuclear localization of E-cadherin has been observed in various types of cancer. However, no information regarding its function in nuclear translocation has been reported. Zhao et al. showed that E-cadherin in the nucleus can be acetylated by CBP at K870 and K871 at the binding domain of the β-catenin promoter, while the acetylation of E-cadherin was reversed by SIRT2 (Fig. 1). Functionally, E-cadherin acetylation attenuates its interaction with β-catenin promoters, increasing downstream gene expression and accelerating EMT in cancer cells [63]. In addition to E-cadherin, vimentin, another EMT marker, is acetylated to facilitate its expression in HCC cells. In addition, SIRT5 binds to vimentin and deacetylates it at K120 in HCC cells [64]. However, whether other epithelioid and mesenchymal markers that are responsible for EMT could be regulated by non-histone acetylation has not been reported to date.

### Non-histone acetylation in modulating EMT-TFs

As described above, an increase in histone acetylation at the promoters of EMT-TFs facilitates their expression and initiates EMT. Interestingly, emerging evidence demonstrates that the acetylation of core EMT-TFs, including Snail, Twist, and ZEB, also contributes to EMT in different cancers [21]. In particular, multiple KATs and KDACs have been found to acetylate EMT TFs to regulate EMT (Fig. 1).

### Snail1 and Snail2

Recent studies from different groups have reported that Snail1 can be acetylated to regulate its function [65–67]. In particular, based on Co-IP and mass spectrometry (MS) analysis, CBP was found to acetylate K146 and K187 of Snail1 to enhance its target gene expression in many cancer cells [68]. P300 acetylates Snail1 at K187 to facilitate its transcription in lung cancer (Fig. 1) [69]. Moreover, Snail1 acetylation contributes to the reduction of ubiquitylation, enhancing its stability [67]. As for Snail2, it has been found that the acetylation of this protein could promote its expression [70], and a study by Dai et al. showed that CBP interacts with Snail2, as detected by Co-IP assay, leading to the acetylation of Snail2 at K166 and K211. Additionally, Snail2 acetylation mediated by CBP can stabilize Snail2 and promote EMT in breast cancer [71]. However, Zhou et al. indicated that Snail2 undergoes acetylation-dependent protein degradation, and SIRT2 deacetylates Snail2 at K116 to prevent Snail2 degradation and extend Snail2 stability in breast cancer cells [72]. The reasons for the inconsistent role of Snail2 acetylation at different lysine residues assessed by different groups in breast cancer cells are not clear. Thus, further investigations are needed to explore the exact effect of Snail2 acetylation at different lysine residues on EMT.

### Twist1 and Twist2

Twist1 is acetylated by PCAF, and Twist1 acetylation promotes its nuclear localization and transcriptional potential to initiate tumorigenesis. Moreover, K73, K76, and K77 in Twist1 in bladder cancer cells [73]. Twist1 is also acetylated by Tip60 at K73 and K76 in basal-like breast cancers (Fig. 1) [74]. However, whether Twist2 can be acetylated remains unclear.

### ZEB1 and ZEB2

ZEB1 and ZEB2 are capable of binding to regulatory gene sequences to repress or activate transcription. Interestingly, PCAF and P300 interact with ZEB1 to switch the protein from a transcriptional repressor to a transcriptional activator [13]. Furthermore, based on Co-IP assay, Mizuguchi et al. found that P300 and PCAF interact and form a complex with ZEB1, leading to its acetylation (Fig. 1). Moreover, acetylated ZEB1 increases miR-200c/141 transcriptional activity to facilitate EMT [75]. However, acetylation sites in ZEB1 were identified by Mizuguchi et al. Until now, whether acetylation affects the function of ZEB2 is not been well-defined.

### Non-histone acetylation in modulating other transcription factors

In addition to established EMT-TFs, other transcription factors have also been shown to regulate EMT. However, the effects of different acetylated transcription factors on the EMT are diverse. For example, transcription factor KLF15 participates in EMT. The acetylation of KLF5 at K369 through P300 that induced by TGF-β maintains EMT and causes the metastasis of prostate cancer cells [76]. Although the acetylated transcription factor YY1 participates in EMT, SIRT1 can inhibit EMT in renal tubular cells by inhibiting YY1 acetylation in diabetic nephropathy [77]. Acetylated ISX and BRD4 are also involved in EMT. Moreover, based on MS analysis, PCAF was found to mediate ISX acetylation at K69 and BRD4 acetylation at K332 to facilitate EMT [78]. In addition, P300 was found to control EMT via the c-Myb acetylation at K471, K480, and K485 (Fig. 1) [79]. As mentioned above, α-SMA is an EMT-related mesenchymal marker. Ding et al. found that acetylated C/EBPβ can interact with the αSMA promoter and facilitate TGF-β-induced transcription of αSMA [80]. SIRT1 reverses C/EBPβ acetylation. Deacetylated C/EBPβ reverses elevated α-SMA expression. However, the acetylation sites of C/EBPβ have not been reported.

In contrast, GATA3 is a limiting factor in EMT. GATA3 acetylation at K119 mediated by CBP contributes to the inhibition of EMT, indicating that the deacetylation of GATA3 has a promotional effect on EMT in lung adenocarcinoma. However, HDAC1, HDAC2 and HDAC3 can deacetylate GATA3 [81]. As a subunit of the transcription factor AP-1, c-Jun, especially deacetylated c-Jun, contributes to the EMT. However, the downregulation of HDAC3 inhibits EMT in cutaneous squamous cell carcinoma by promoting c-Jun acetylation [82]. HIF-1α is also a regulator of EMT, and the deacetylation of HIF-1α, but not its acetylation, benefits EMT. Moreover, dependent on HDAC4, 14-3-3ζ was found to upregulate the expression of HIF-1α by promoting its deacetylation, which was detected by co-IP assays to facilitate EMT in HCC [83]. However, the acetylation sites of HIF-1α have not been identified. Zhu et al. also found that MTA2 enhances HIF-1α stability via deacetylation by interacting with HDAC1 to further activate HIF-1α transcriptional activity (Fig. 1) [84]. SIRT1 has been shown to have an inhibitory effect on EMT in ovarian cancer cells, with its inhibition of EMT being found to be related to the upregulation of CLDN5, an epithelial marker. Zhang et al. also found that SIRT1 could deacetylate the transcription factor KLF4 to activate CLDN5 transcription and inhibit EMT [85]. However, the deacetylation sites in KLF4 that are regulated by SIRT1 remain unknown. Together, these studies show that deacetylation of certain transcription factors also benefits EMT.

### Non-histone acetylation in modulating signal transduction molecules in different signaling pathways to facilitate EMT

As described in the introduction, multiple signaling pathways participate in the initiation of EMT. Current evidence indicates that acetylation of the core signal transduction molecules is responsible for the activation of these target signaling pathways and the subsequent induction of EMT. To date, many signal transduction molecules in the TGF-β/Smad, NF-κB, JAK/STAT, and PI3-K signaling pathways have been acetylated (Fig. 1). In addition, the acetylation or deacetylation of these signal transduction molecules plays an essential role in the activation of different signaling pathways to induce EMT.

### TGF-β/Smad signaling pathway

The activation of the TGF-β/Smad signaling pathway mediated by TGF-β family receptors plays a critical role in the induction of EMT [4]. Previous studies have focused on the regulation of EMT by the acetylation of important signal transduction molecules in this signaling pathway. In particular, based on Co-IP assay, TGF-β1-mediated EMT has been shown to be associated with Smad2, Smad3, and Smad4 acetylation (Fig. 1) [86, 87]. TGF-β2 promotes EMT by supporting Smad2 acetylation [88]. More importantly, among KATs and KDACs, P300/CBP mediates EMT by acetylating Smad2 and Smad3 [86]. However, SIRT6 inhibits the acetylation of Smad3 [89]. In addition, SIRT1, as well as SIRT7 can lead to an increase in the deacetylation of Smad4 [87, 90]. However, the acetylation sites of different Smad molecules have not been identified.

### NF-κB signaling pathway

Current evidence has demonstrated that increased acetylation of p65 is responsible for NF-κB activation, which also facilitates EMT. For example, Huang et al. showed that trichostatin A (TSA) can promote EMT in esophageal squamous cell carcinoma by inducing the acetylation of p65 at K310 [91]. In addition, the interaction of p65 with P300 facilitates p65 acetylation, as detected by Co-IP experiments, which in turn interacts with the promoter of ZEB1, resulting in a decrease in E-cadherin [92]. However, Astragaloside IV inhibited glucose-induced EMT in podocytes by decreasing p65 acetylation. Similarly, SIRT1 contributes to p65 deacetylation in glucose-induced podocyte EMT, whereas OPN can inhibit SIRT1 expression and promote p65 acetylation in non-small cell lung cancer cells. Conversely, the overexpression of SIRT1 can restrict p65 acetylation, activation, and EMT [93, 94]. In addition, N-Myc-interacting protein (NMI) is capable of promoting the interaction of p65 with HDAC1, HDAC2, and HDAC3 and negatively regulates EMT by inhibiting p65 acetylation (Fig. 1) [95]. Except for p65, this signaling pathway is composed of a variety of signal transduction molecules, including IκB-α, IκB-β, IKKα, and IKKβ [96]. However, the information on the acetylation of these signal transduction molecules is not available.

### JAK/STAT signaling pathway

The activation of the JAK/STAT signaling pathway mediated by cytokine receptors has also been shown to participate in EMT [3, 4]. In particular, STAT3 acetylation induced by IL-6 is required for EMT in CRC cells [97]. P300-dependent STAT3 acetylation, examined by the Co-IP assay, is necessary for EMT in renal tubular epithelial cells (Fig. 1) [98]. In addition, nicotinamide adenine dinucleotide (NAD) is known to have a significant effect on all aspects of human life. However, decreased NAD levels induce STAT3 acetylation to facilitate its activation [99]. Until now, the acetylation sites in STAT3 that are modulated by different factors in various cell types have not been well identified. To date, it remains unclear whether other signal transduction molecules in the JAK/STAT signaling pathway, including JAK1, JAK2, STAT1, and STAT2, can be acetylated and participate in EMT.

### PI3-K signaling pathway

The activation of the PI3-K signaling pathway, which is stimulated by receptor tyrosine kinases, contributes to sensitization of the NF-κB signaling pathway to facilitate EMT [4]. AKT1 is a core signal transduction molecule in the PI3-K pathway, and its activation is dependent on phosphorylation. An et al. showed that HDAC8 can bind to AKT1 to decrease its acetylation while increasing its phosphorylation (Fig. 1). A detailed investigation based on MS suggested that K426 in the AKT protein is the key amino acid residue for the HDAC8-modulated deacetylation of this protein. Moreover, deacetylated AKT stabilizes Snail1 and activates EMT in breast cancer cells [100].

### Targeting protein acetylation or deacetylation as a potential strategy to restrict EMT

Because lysine acetylation or deacetylation relies on KATs and KDACs, and up to now, a variety of small molecule inhibitors have been developed to suppress the catalytic reaction of KATs or KDACs, resulting in the hyperacetylation or deacetylation of histones or non-histone proteins [25, 101]. Our review suggests that the acetylation of histones and non-histone proteins facilitates EMT. In addition, the deacetylation of certain transcription factors and signal transduction molecules also contributes to EMT. Therefore, it is not surprising that KAT and KDAC inhibitors can suppress EMT.

To date, few KAT inhibitors have been investigated for EMT inhibition (Table 2). Current studies show that anacardic acid, a major constituent of cashew nutshells, is a non-selective KAT inhibitor [102, 103], and garcinol, a polyisoprenylated benzophenone derivative, mainly targets P300 [104] and ICG-001, a small molecule that inhibits CBP/β-catenin [105]. EGCG, a polyphenolic component from the green tea, is an inhibitor with the ability to suppress P300/CBP activity, blocking EMT in different cancer cells [86, 106]. In the future, further KAT inhibitors will need to be evaluated to determine their precise roles in the restriction of EMT in various cancer cells.

**Table 2:** The effect of KAT and KADC inhibitors on EMT in different cancer cells

Drug name	Drug types	KAT or HDAC specificity	Target cancer cells	The role on EMT	References
Anacardic acid	KAT inhibitor	Non-selective KAT inhibitor	Breast cancer, Prostate cancer	Inhibit	[102, 103]
Garcinol	KAT inhibitor	P300 inhibitor	Breast cancer	Inhibit	[104]
EGCG	KAT inhibitor	P300/CBP inhibitor	Lung cancer	Inhibit	[86, 106]
ICG-001	KAT inhibitor	CBP/β-catenin inhibitor	Nasopharyngeal carcinoma	Inhibit	[105]
Mocetinostat	HDAC inhibitor	class I KADC inhibitor	Pancreatic cancer, Lung cancer	Inhibit	[106]
SAHA	HDAC inhibitor	class I, II and IV, KADC inhibitor	Head and neck cancer, Triple-negative breast cancer, Breast mesenchymal cancer	Inhibit	[106–109]
TSA	HDAC inhibitor	class I, II and IV, KADC inhibitor	Breast cancer, Lung cancer	Inhibit	[110, 111]
Sodium butyrate	HDAC inhibitor	Class I, II KADC inhibitor	HCC, Colorectal cancer, Bladder cancer	Inhibit	[112–114]
VPA	HDAC inhibitor	Class I, II KADC inhibitor	Gastric Cancer, HCC, Prostate carcinoma, Renal cell carcinoma, Esophageal squamous cell carcinoma, Prostate carcinoma	Inhibit	[115–120]
MS-275	HDAC inhibitor	Class I KADC inhibitor	Breast cancer, Non-small cell lung cancer	Inhibit	[121–123]
LBH589	HDAC inhibitor	pan-KADC inhibitor	Colorectal cancer, Breast cancer, Prostate cancer, HCC	Inhibit	[124–128]
TSA	HDAC inhibitor	class I, II and IV, KADC inhibitor	Esophageal squamous	Activate	[129]
SAHA	HDAC inhibitor	class I, II and IV, KADC inhibitor	Prostate cancer, Lung cancer, Gastric cancer, Triple negative Breast cancer	Activate	[45, 70, 130, 131]
VPA	HDAC inhibitor	Class I, II KADC inhibitor	Colorectal cancer, Triple negative breast cancer, Breast cancer, HCC, Colon carcinoma	Activate	[66, 132–135]

Several HDAC inhibitors, including belinostat, vorinostat, and panobinostat, have been approved for the treatment of cancers [25]. Furthermore, different clinical trials assessing the role of HDAC inhibitors in cancer treatment are underway [25]. More importantly, studies from different research groups indicate that the effect of HDAC inhibitors on the inhibition of various cancer cells is mainly mediated by targeting EMT (Table 2). For example, mocetinostat, a type of benzamide, is a class I HDAC inhibitor [107], while suberoylanilide hydroxamic acid (SAHA) and TSA, a type of hydroxamic acid [106, 108–111], are class I, II, and IV HDAC inhibitor. Valproic acid (VPA) and sodium butyrate, two types of short-chain fatty acids [112–120], are class I and II HDAC inhibitors, while MS-275, a type of benzamide, [121–123], is a class I HDAC inhibitor, and LBH589, a hydroxamic acid derivative, is a pan-KADC inhibitor [124–128]. These have all been identified to suppress EMT in different cancer cells.

To date, the exact molecular mechanisms that contribute to the inhibition of EMT mediated by these HDAC inhibitors have yet to be well defined. Among these HDAC inhibitors, TSA [129], SAHA [45, 70, 130, 131], and VPA have been shown to facilitate EMT in various cancer cells [66, 132–135]. The reasons for these contradictory conclusions regarding the effects of these three HDAC inhibitors on EMT remain unclear. Molecular factors involved in EMT are key targets for the development of new therapeutic strategies for tumor suppression. As mentioned above, many factors, including EMT-TFs, EMT-related lncRNAs, and EMT-related signalling pathways, are involved in the regulation of EMT. However, to date, the exact molecular targets of different HDAC inhibitors in various tumor types remain largely unknown. Therefore, further studies are needed to confirm the precise targets and detailed mechanisms of the effects of HDAC inhibitors on EMT in various types of cancers.

## Conclusions

In summary, as a complex biological trans-differentiation process, EMT not only contributes to metastasis and invasion but also induces cell stemness and drug resistance in various cancers. In addition to EMT, partial EMT or hybrid E/M states are also present in tumors [9, 10]. Accumulating evidence indicates that tumor progression and metastasis are favored by tumor cells in partial EMT or hybrid E/M states [10]. It should be noted that despite our understanding of the role and mechanisms related to EMT, partial EMT, or hybrid E/M states in cancer progression is growing, much still remains to be elucidated. As mentioned above [15, 18, 19, 21], EMT could be regulated by epigenetic modulation and PTM. Drugs that influence epigenetic factors, including DNA methylation and histone modification, or target different types of PTMs, such as ubiquitylation, phosphorylation, and sumoylation, are potential therapeutic approaches for overcoming EMT. However, the molecular mechanisms underlying the epigenetic and post-translational mechanisms that modulate EMT are complex. Thus, to better target EMT in clinical treatment, a more thorough understanding of the cellular factors involved in epigenetic modulation and PTM is required.

Our review indicates that histones and non-histone proteins related to EMT can be acetylated. However, the effects of acetylation on these two proteins are diverse. Histone acetylation is an important epigenetic regulator, whereas non-histone protein acetylation is a critical PTM. Therefore, a better understanding of both the histone acetylation and non-histone acetylation of EMT-related cellular factors is important to develop a workable strategy to delete cells that have undergone EMT in cancer treatment. EMT-TFs, including Snail, Twist, and ZEB, are activated early to initiate EMT. Because of their vital role in modulating the EMT process, inhibiting their expression may be a highly effective way to reverse this process. In addition, many EMT-related signaling pathways, such as the TGF-β/Smad, NF-κB, JAK/STAT, and PI3-K signaling pathways, are known to contribute to the production of the EMT phenotype; therefore, inhibitors of these pathways need to be developed to overcome EMT. Our review of the literature shows that the expression and activation of these transcription factors and signaling pathways can be modulated by histone or non-histone protein acetylation. These findings imply that targeting histone or non-histone protein acetylation is an ideal strategy for controlling these transcription factors and signaling pathways, thereby inhibiting EMT.

More importantly, the acetylation of histone or non-histone proteins is controlled by KATs, and accumulating evidence indicates that KAT inhibitors can effectively inhibit EMT. However, the molecular targets mediated by KAT inhibitors that facilitate EMT suppression have not been fully investigated. In addition to acetylation, the evidence presented here shows that the histone deacetylation of EMT-related epithelioid markers and the non-histone deacetylation of certain transcription factors and signal transduction molecules also contribute to EMT. The deacetylation of histone and non-histone proteins is mainly controlled by KADCs, and the use of KADC inhibitors may provide an effective treatment strategy for EMT. Therefore, further identification of the specific functions and exact mechanisms of histone and non-histone protein acetylation and deacetylation linked to EMT mediated by KATs or KADCs may provide a unique opportunity to target the EMT-associated cancer progression.

## Data Availability

Not applicable.

## Abbreviations

EMT: Epithelial-mesenchymal transition
KATs: Lysine acetyltransferases
KDACs: Lysine deacetylases
EMT-TFs: EMT-inducing transcription factors
PTMs: Post-translational modifications
HATs: Histone acetyltransferases
HDACs: Histone deacetylases
CPEΔN: N-terminal truncated carboxypeptidase E
LncRNA: Long non-coding RNA
HCC: Hepatocellular carcinoma
GHET1: Gene gastric carcinoma high expressed transcript 1
TINCR: Terminal differentiation-induced non-coding RNA
PLAC2: Placenta-specific protein 2
SAHA: Suberoylanilide hydroxamic acid
VPA: Valproic acid
TSA: Trichostatin A
